# Relationships among Sports Group Cohesion, Psychological Collectivism, Mental Toughness and Athlete Engagement in Chinese Team Sports Athletes

**DOI:** 10.3390/ijerph19094987

**Published:** 2022-04-20

**Authors:** Song Gu, Lan Xue

**Affiliations:** College of Physical Education and Health Sciences, Zhejiang Normal University, Jinhua 321004, China; xl@zjnu.cn

**Keywords:** psychological collectivism, cohesion, athlete engagement, mental toughness

## Abstract

Background: Cohesion is an important factor affecting sports performance. This study constructed a mediating model to explore the mechanism of cohesion toward psychological collectivism, mental toughness, and athlete engagement of Chinese team sports athletes, and to investigate the mediating effect of psychological collectivism and mental toughness on cohesion and athlete engagement. Methods: A total of 326 active Chinese athletes (54% males, 46% females) aged 14 to 26 years (M = 19.63, SD = 6.51) from eight sports were investigated by questionnaire. Results: The athlete engagement can be predicted significantly and positively by cohesion and its dimensions, and ATG-T is more important in advantage analysis. Direct and indirect paths indicate that cohesion affects athlete engagement, through the mediating effects of psychological collectivism, the mediating effects of mental toughness, the serial multiple mediating of psychological collectivism and mental toughness. The mediating effect model had a satisfactory goodness of fit and explained 50.5% of the variance in athlete engagement, and the SEM revealed the mechanism of cohesion in Chinese athlete engagement to a certain extent. Conclusion: Psychological collectivism is the embodiment of high-quality cohesion in Chinese team sports. The increase in cohesion and psychological collectivism can improve Chinese athletes’ ability to cope with stressful situations in sports, which may allow them to achieve a better performance through athlete engagement.

## 1. Introduction

For many sporting experiences, athletes are members of groups or teams. These groups have a strong effect on the members of the group. Cohesion is one of the most important small group variables that is derived from the evolution of group culture [1]. Although previous studies have shown that group cohesion can improve athletes’ sports performance [2,3], a lack of discussion on the mechanism and conditions between the two was observed because objective performance is affected by many factors [4]. In addition, in a large sample survey, the competition results of various events are also difficult to integrate into a unified scale. Therefore, measuring the psychological variables that reflect the sports performance at the individual level has become an efficient strategy for cohesion research. The purpose of this study was to investigate the relationships among cohesion psychological collectivism, mental toughness, and athlete engagement, in addition to gaining an in-depth understanding of the relationship between cohesion and sports performance from a positive psychology perspective.

## 2. Theoretical Background and Hypotheses

Cohesion is one of the most important small group variables, which reflects the integration and coordination level of the group and is an important internal factor affecting the realization of the goal [1]. Cohesion is an important source of the athletes’ social support [1], but it is also positively associated with a variety of group and individual outcomes, which comprise team performance, effectiveness, confidence, positive affect, and exercise adherence [2,3,4]. The generally accepted definition is that cohesion is a dynamic process that is reflected in the tendency of a group to stick together and remain united in the pursuit of its instrumental objectives and/or for the satisfaction of member affective needs [1]. Cohesion should be divided into at least two aspects: task cohesion, which is related to the commitment of team objectives and achievement indicators; and social cohesion, which involves more interpersonal relationships, such as friendship and emotional support [1].

The relevant results of team cohesion have provided a good theoretical basis for studying sports team cohesion. The analysis of the relationship between sports team cohesion and sports performance is based on the basic analysis framework of team cohesion [2]. After Carron and others defined the operational definition of cohesion, four dimensions were formed: individual attractions to the group—task (ATG-T), individual attractions to the group—social (ATG-S), group integration—task (GI-T), and group integration—social (GI-S), which were quickly applied to the research of sports team cohesion and achieved a large number of relevant results [1,4].

The relationship between cohesion and sports performance has always been the focus of attention [2,4,5]. However, research shows that the actual situation is much more complex. The sports involved in the research of positive correlation between cohesion and sports performance are mainly collective events, such as basketball, volleyball, and hockey [4,5], whereas a small number of sports involved in the research of a negative correlation or no correlation are individual events, such as bowling and shooting [4,5]. Because of the complexity itself and our the limitation of our understanding, a lack of discussion about the mechanism and conditions between cohesion and sports performance was observed.

Recent studies have identified the positive effects of cohesion on engagement in many fields and environments, such as employment [6], classroom [7], community [8], and army [9]. Relevant studies mostly take engagement as the observation index of the work performance of the organization members. In organizational behavior research, engagement refers to the situation in which the organization members are in a lasting and perfect state full of positive emotions and motivation [10]. Lonsdale (2006) introduced the concept into the field of sports, and determined that athlete engagement is the perfect link connecting individual characteristics, sports factors, and sports performance [11]; compared with sensory indicators such as sports satisfaction, athlete engagement can intuitively reflect the positive experience of individual cognition, and behavior, self-confidence, dedication, vigor, and enthusiasm are its main characteristics [11]. The influencing factors of athlete engagement comprise internal factors such as basic psychological needs, gratitude, mental toughness, and coping style, in addition to external factors such as the coach–athlete relationship, motivation atmosphere, and social support. Zhang (2011) believes that athlete engagement is an important indicator of the athletes’ positive psychology, and can reflect the athletes’ positive and healthy psychological state, which is conducive to stimulating the athletes’ positive qualities, such as optimism, resilience, sense of significance, and creativity, to promote the development and sophistication of athletes, in addition to laying a solid foundation for enhancing their sports ability and improving their sports performance. Therefore, athlete engagement may be used as an alternative index of sports performance to appropriately reflect the mechanism of cohesion in individual sports performance.

At present, the research on the influencing factors and mechanism of athlete engagement is limited. By looking at the variables for this study, cohesion is considered within the motivation atmosphere and social support. One study found that team cohesion was positively related to organizational identity and work engagement [12]. As a special organizational identity, psychological collectivism may be consistent with the perspective of this study and fit under the basic psychological needs and gratitude factors for the athlete engagement.

Recent studies have found young athletes who are closely related to their own sports teams can perceive more cohesion [13], which seems to be promoted by the existence of collective-oriented team members [14,15]. In organizational behavior, a large number of studies cited collectivism as an important feature of a cohesive group [16]. Historically, collectivism has mostly been constructed as a cultural variable, representing the overall model in a complex society [17,18]. This approach is mainly due to Hofstede’s (1980) cross-cultural research, which identified a range of differences focusing on the value system related to national work. However, this perspective operates collectivism as a social preference by using the national average score, which cannot accurately explain or attempt to explain individual behavior. In recent years, scholars have constantly proposed different views and advocated that collectivism should be regarded as an individual difference variable in the team environment [19]; that is, the analysis from the culture to the personality. This specific perspective is considered to accurately reflect the collectivist tendency in the individual’s basic psychological process [20], which is also known as psychological collectivism.

Psychological collectivism refers to individuals who regard themselves as members of one or more groups, who are mainly inspired by the norms of members of the group, give priority to the goals and well-being of members of the group, and emphasize their connection with members of other groups [20]. From this perspective, Jackson et al. developed a corresponding questionnaire that comprises preference, reliance, concern, acceptance, and goal priority [20]. The measurement demonstrates that psychological collectivism is closely related to several important personal variables of staff in the team environment, such as member dependence, emotional, information and evaluation support, effective team operation behavior, and citizenship behavior [21,22,23]. In addition, the harmony and cohesion of the group may affect the individual emotion in a high level of psychological collectivism [22]. Recently, this relevance has also been recognized in elite sports situations [24]. Considering the link between collectivism and supportive team behavior, athletes participating in these environments should experience more social support and obtain subsequent persistence behavior.

Competitive sports events are full of pressure and challenges. The athlete engagement promoted by cohesion and psychological collectivism aims not to eliminate the existence of negative mentality in competitive sports events, but to ensure the targeted engagement of athletes by improving individual toughness. Mental toughness is the quality that reflects the athletes’ self-confidence, focus, and motivation in stressful situations; it refers to a psychological advantage inherited or developed after birth [25], and self-confidence, control, and constancy are its main characteristics [26]. Research has found that athletes with high mental toughness have high control beliefs about stress situations, tend to evaluate stresses as challenges rather than threats, have high coping self-efficacy, and adopt more problem-focused coping strategies [26]. Athletes with this advantage may be better able to deal with the pressure in competition, training, and life than their competitors, be more firm, focused, and confident in a pressure situation, and maintain self-control, to perform better than their competitors [27]. Therefore, mental toughness significantly improves the individual’s sports state, wherein athletes rarely experience the psychological and physiological discomfort that limits the exertion of personal ability [25]. In addition to the protective effect in stress situations, the study found that mental toughness also has obvious advantages. For example, it can enhance the athletes’ optimism, flow experience, and self-determination motivation, and improve competition results [25,26,27]. Gucciardi et al. [28] found through qualitative research that athletes with high mental toughness will not only have greater individual positive cognition, but also devote themselves to training and competition with positive attitudes, such as task focus, active self-discipline, and perseverance. In addition, Wang (2014) found that mental toughness significantly negatively predicts athletes’ burnout, whereas athlete engagement, as the opposite of burnout, may be positively affected by mental toughness.

In terms of the literature review, we found that the development of mental toughness involves several unique mechanisms that operate together over a long period of time and through unique developmental stages [25]. In addition to psychological skills and strategies, features relating to the motivational climate, external assets (i.e., coaches, peers, parents, senior athletes, sport psychologists, team-mates) [26,27,28,29], and both sport and non-sport related developmental experiences were discussed as the most important mechanisms. Jones (2006) summarized a mental toughness theory and divided the process of mental toughness into three stages: mental toughness behavior is controlled by mental toughness thinking, and mental toughness thinking is influenced by environment and personality. By looking at the variables of this study, cohesion and psychological collectivism falls under the mental toughness environment, including the motivation climate and external assets, whereas athlete engagement can fit under mental toughness behavior. Similar findings revealed that cohesion can significantly affect the formation of mental toughness with a sport-specific sample of college athletes [26].

Based on the above discussion, cohesion, psychological collectivism, mental toughness, and athlete engagement indicate that many different constructs and relationships warrant further examination. First, athlete engagement can be used as an alternative index of sports performance to reflect the psychological mechanism of cohesion affecting sports performance. Second, many studies cited collectivism as an important feature of a cohesive group. However, there is a lack of empirical research. This study attempts to demonstrate the relationship between sports group cohesion and psychological collectivism, and the impact of psychological collectivism on sports performance/athlete engagement. Third, there are mediating factors in the influence of cohesion and psychological collectivism on athlete engagement, which need the help of stress resistance ability. Based on the above, these research hypotheses are proposed:

**Hypothesis** **1** **(H1).** *Cohesion and its constructs are positively related to athlete engagement.*

**Hypothesis** **2** **(H2).** *Psychological collectivism mediates the relationship between cohesion and athlete engagement.*

**Hypothesis** **3** **(H3).** *Mental toughness mediates the relationship between cohesion and athlete engagement.*

**Hypothesis** **4** **(H4).** *Psychological collectivism and mental toughness sequentially mediate the relationship between cohesion and athlete engagement.*

## 3. Methods

### 3.1. Recruitment and Participants

The participants were professional athletes and high-level college athletes selected from the national training team and Zhejiang, Heilongjiang, Liaoning, and other provinces and cities in China. All of them are team sports athletes having at least three years of sports training experience. Considering that *n* = 200 is the minimum sample size for SEM [30], a total of 445 athletes were investigated by questionnaire in this study, after deleting questionnaires with an overly short response time (less than three min) and those with answers that tend to be consistent, 326 were effectively recovered, with an overall effective rate of 73.3%.

### 3.2. Instruments

The questionnaire comprised three parts. First, we stated that this survey was being conducted voluntarily and anonymously. The answers to the questionnaire were only available for the researchers and not for commercial or any other use. Second, collection of the athletes’ basic information. Third, the scale of the questionnaire used in this study. All questionnaire responses involved in this study were scored in five-point Likert scales, from strongly disagree (1) to strongly agree (5); the higher the score, the higher the recognition and acceptance of the item. The details are as follows:

#### 3.2.1. Group Environment Questionnaire (GEQ)

The GEQ compiled by Carron (2010) and translated by Ma Hongyu [31] was adopted. This questionnaire is a special measurement questionnaire for sports cohesion, which has good reliability and validity in Chinese use. There are 15 items in the questionnaire, including four dimensions, ATG-T, ATG-S, GI-T and GI-S, that respectively represent the two levels of integration and involvement of task cohesion and social cohesion. In this study, the total amount and each dimension’s Cronbach’s α is 0.71~0.87.

#### 3.2.2. Psychological Collectivism Questionnaire (PCQ)

The PCQ compiled by Jackson (2006) and translated by Zhang Lan [32] was adopted. This questionnaire has passed many research tests in China, and has a total of 15 items, including five dimensions: preference, reliance, concern, acceptance, and goal priority. According to the purpose of the study, this questionnaire describes and supplements the content of sports situations. For example: “I can accept the rules and regulations of the team” → “I can accept the rules and regulations of the sports team”, “compared with my personal work goal, the team task goal is more important” → “compared with my personal sports goal, the team sports goal is more important”, etc. In this study, the total amount and each dimension’s Cronbach’s α is 0.74~0.91.

#### 3.2.3. Sports Mental Toughness Questionnaire (SMTQ)

The SMTQ was compiled by Sheard et al. (2009) and translated by Wang Bin et al. There are 12 items in total, including three dimensions of self-confidence, constancy, and control. In this study, the total amount and each dimension’s Cronbach’s α is 0.71~0.82.

#### 3.2.4. Athlete Engagement Questionnaire (AEQ)

The AEQ was compiled by Lonsdal et al. (2007) and revised by Ye Lv’s translation [33]; there are 16 items, including four dimensions of self-confidence, dedication, vigor, and enthusiasm. In this study, the total amount and each dimension’s Cronbach’s α is 0.90~0.95.

### 3.3. Data Collection

Paper-and-pencil self-administered questionnaires were distributed to athletes after the end of training. To ensure the quality of responses, the research assistants read the instructions and explained the purposes and requirements of the questionnaire at the beginning. Completion of the survey took an average of seven minutes.

### 3.4. Validity and Reliability of the Instrument

The reliability and validity of the measurement scale were subsequently evaluated. Confirmatory factor analysis (CFA) was used to establish the internal validity of each construct. CFA showed that the modified model fit the data well: CMIN/DF = 3.68, RMSEA = 0.06, GFI = 0.91, NFI = 0.92, CFI = 0.94. Then, the internal consistency reliability and composite reliability (CR) were evaluated (shown in Table 1). The Cronbach’s α values of each construct were all above 0.7, thus indicating an acceptable reliability [30]. Moreover, the CR value of each construct surpassed 0.7, thus showing good composite reliability [30]. Regarding the convergent validity, it can be evaluated by average variance-extracted (AVE) and factor loadings (FL). The values of AVE and FL of each construct were higher than 0.4, thus indicating an acceptable level of convergent validity (see Table 1) [30].

### 3.5. Common Method Bias

The data for all constructs were collected simultaneously through a self-reporting questionnaire; thus, the common method bias (CMB) was a potential problem. To reduce the interference of common method bias on validity, this study applied a balanced item order, anonymous questionnaire measurement, and standardized measurement in the process of the questionnaire. CMB can be tested by Harman’s one-factor test and the CFA marker variable approach [30]. Results show that the variance explained by the first factor of principal component analysis was 35.23%, which was less than the critical standard of 40%. Confirmatory factor analysis found that the fitting index of the 16-factor model (χ^2^ = 180.51, χ^2^/df = 3.68, RMSEA = 0.06, CFI = 0.94, GFI = 0.91, NFI = 0.92) is significantly better than the single-factor model (χ^2^ = 531.53, χ^2^/df = 10.52, RMSEA = 0.12, CFI = 0.55, GFI = 0.53, NFI = 0.52).

### 3.6. Data Analysis

SPSS 22.0 (IBM, Armonk, NY, USA) was used to input the questionnaire data for descriptive analysis, reliability analysis, and hierarchical regression analysis; AMOS 21.0 (IBM, Chicago, IL, USA) was used for a common method deviation test, confirmatory factor analysis, and mediation effect model analysis.

## 4. Results

### 4.1. Descriptive Analysis

A total of 445 questionnaires were distributed from 1 June to 1 October 2020. After deleting questionnaires with an overly short response time (less than three min) and those with answers that tended to be consistent, 326 were effectively recovered, with an overall effective rate of 73.3%. Among them, 175 were male (53.7%) and 151 were female (46.3%); there were 72 state second-class athletes (22.1%), 127 state first-class athletes (40.0%), 76 state elite athletes (23.3%), and 52 people who lacked sports class information. The average age of athletes was 19.63 years (SD = 6.51), and the average training period was 6.78 years (SD = 3.37). Sports comprised basketball (74), volleyball (36), football (63), cricket (47), ice hockey (20), curling (11), group aerobics (47), and others (28).

### 4.2. Correlational Analysis

Table 2 illustrates the mean (M) and standard deviations (SD) of cohesion, psychological collectivism, mental toughness, and athlete engagement, and Spearman analysis was used to examine the correlation coefficients among cohesion, psychological collectivism, mental toughness, and athlete engagement. Results show that all variables are significantly correlated, and the correlation coefficient ranged from 0.35 to 0.67, thus supporting the effectiveness of the overall data of the measurement model and the rationality of the topic packaging strategy.

### 4.3. Analysis of the Advantages of Different Dimensions of Cohesion in Predicting Athlete Engagement

After controlling for the gender, age, training years, and sports grade, cohesion can explain 28% of the variation of athlete engagement alone by using a hierarchical regression analysis (*β* = 0.62, *t* = 7.08, *p* < 0.001). However, the potency of various dimensions of cohesion on athlete engagement is unclear. To clarify the interpretation effect of relevant dimensions, the advantage analysis method with model independence was used to calculate the change value of *R*^2^ after each explanatory variable was added to the sub-model without the variable itself, to explain the relative contribution of each dimension of cohesion to the effect of athlete engagement. Results show that (Table 3) all dimensions of cohesion are significantly and positively correlated with athlete engagement, and H1 is verified; among them, ATG-T (40.3%) contributed the most to the explained variation.

### 4.4. Mediating Effect of Psychological Collectivism and Mental Toughness in Cohesion and Athlete Engagement

**Figure 1:**
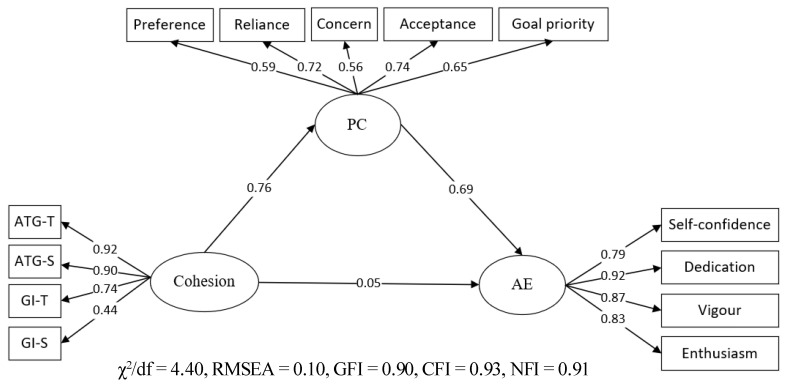
Mediating role of psychological collectivism.

In the mediating effect model (Figure 1), cohesion significantly predicted psychological collectivism (PC) (*β* = 0.76, *p* < 0.01) and psychological collectivism significantly predicted athlete engagement (AE) (*β* = 0.69, *p* < 0.01), indicating that an indirect effect exists on the path of psychological collectivism in cohesion and athlete engagement, and the effect value is 0.76 × 0.69 = 0.52; thus, H2 is verified.

**Figure 2:**
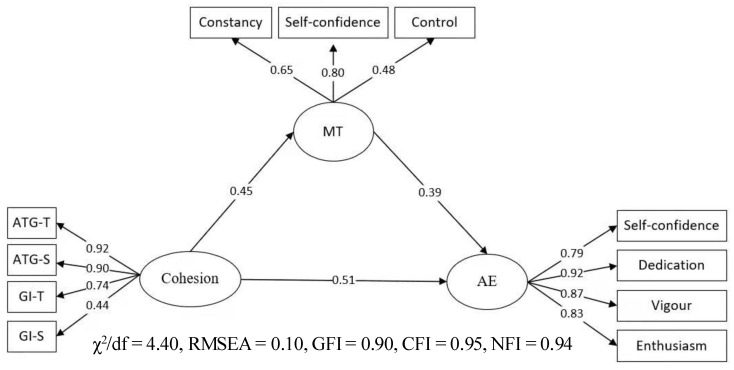
Mediating role of mental toughness.

In the mediating effect model (Figure 2), cohesion significantly predicts mental toughness (MT) (*β* = 0.45, *p* < 0.01) and mental toughness significantly predicted athlete engagement (*β* = 0.39, *p* < 0.01), indicating that there is an indirect effect on the path of mental toughness in cohesion and athlete engagement, and the effect value is 0.45 × 0.39 = 0.18; thus, H3 is verified.

**Figure 3:**
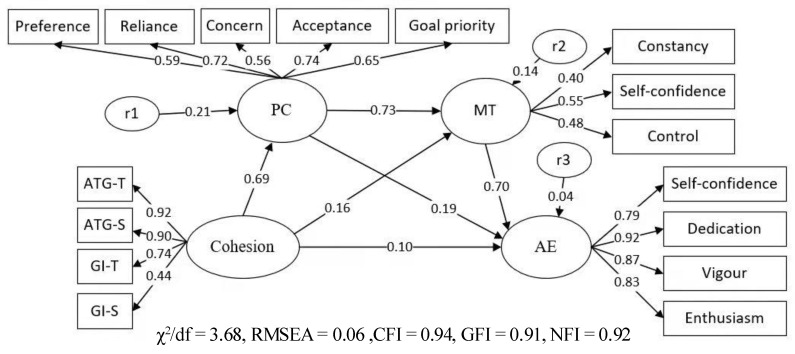
Mediating role of psychological collectivism and athlete engagement.

In the sequential mediating effect model (Figure 3 and Table 4), cohesion significantly predicted psychological collectivism (*β* = 0.69, *p* < 0.01), mental toughness (*β* = 0.16, *p* < 0.01), and athlete engagement (*β* = 0.10, *p* < 0.01); psychological collectivism significantly predicted mental toughness (*β* = 0.73, *p* < 0.01) and athlete engagement (*β* = 0.19, *p* < 0.01); mental toughness significantly predicted athlete engagement (*β* = 0.70, *p* < 0.01), indicating that an indirect effect exists on the path of psychological collectivism in cohesion and athlete engagement, an indirect effect exists on the path of mental toughness in cohesion and athlete engagement, and an indirect effect exists on the path of psychological collectivism and mental toughness in cohesion and athlete engagement, and its effect value is 0.69 × 0.73 × 0.70 = 0.35; thus, H4 is verified. Among them, the total indirect effect of psychological collectivism and mental toughness accounted for 85.5%, which shows that the intermediary effect is greater than the direct effect in the effect of cohesion on athlete engagement. Through the model fit’s *R*^2^ calculation, cohesion, psychological collectivism, and mental toughness explained 50.5% of the variation in athlete engagement.

## 5. Discussion

### 5.1. Direct Effect of Cohesion on Athlete Engagement

This study found that there was a significant positive correlation between cohesion and athlete engagement, and cohesion can also independently and significantly predict athlete engagement in regression model. This result shows that, in the field of competitive sports, cohesion as an important external resource that can significantly improve the athletes’ engagement and promote them to devote themselves to sports with full enthusiasm, vitality, and great self-confidence. In sports, cohesion is an important source of social support for athletes [2]. When athletes have more social support, they experience a higher sense of belonging and security, and be able to obtain effective suggestions and guidance from others to improve their sport skills, so as to improve their competence and self-confidence. Thus, a good cohesive environment undoubtedly plays a significant role in promoting the satisfaction of basic psychological needs, the stimulation of internal motivation, and the improvement of engagement.

Carron (2010) indicated that the development of sports cohesion includes three conditions: first, the group goal is clear and is recognized by members. Second, the needs, motives, and emotions among members are fully understood and supported. Third, those with prestige form the backbone, which plays the role of regulating and communicating interpersonal relations, decision making, organization, and leadership. When these conditions are established, the team can produce the energy amplification effect of mutual encouragement and improving behavior efficiency, while achieving the self-defense effect of mutual protection of external pressure [1,2,3,4,5]. These gain effects can stimulate the athletes’ self-determination motivation [34], and continue to influence members to promote them to maintain an outstanding state and unyielding personality performance.

In previous studies, inconsistent research results have been observed regarding the relationship between cohesion and sports performance. Ma Hongyu (2002) and others believe that this is related to the individual/collective sports of cohesion and the goal setting of the group [4]. Considering these effects, this study selected team sports athletes as subjects. Through the advantage analysis, the four dimensions of cohesion can significantly and positively predict athlete engagement and play their own unique roles. According to the demand resource model theory, the autonomy task goal and social support can inhibit fatigue and improve the members’ engagement [35]. In addition, the results may also be related to the cultural values of Chinese athletes. Some Chinese scholars believe that Chinese athletes have stronger feelings of family and country, sense of responsibility, and relationship needs [36]. They are more likely to project the purpose, tasks, and principles of group activities onto the standards of individual behavior, and automatically adjust and adapt to the behavior norms determined by these indicators [36]. The advantage analysis also shows that, compared with the other dimensions, ATG-T is an important factor affecting athlete engagement. This is similar to the previous results on the relationship between cohesion and sports performance.

### 5.2. The Intermediary Role of Psychological Collectivism and Mental Toughness between Cohesion and Athlete Engagement

In addition to the direct effect, cohesion can also affect athlete engagement through three indirect paths:

Cohesion has a positive effect on athlete engagement through psychological collectivism. This result shows that psychological collectivism is not only an important embodiment of a cohesive group, but also a psychological process in which cohesion promotes the performance of team sports. Strong and stable cohesion can not only enhance group control and urge members to exert every effort to achieve their goals, but also help to strengthen the belief of group members to win and enhance cooperation [1]. According to the self-classification theory [37,38], the improvement in the saliency of internal and external group classification may enhance the consistency and similarity between self and internal group members, and eventually leads to the change in many social identity effects of individual perception. This is similar to the preference for internal groups, the exclusion of external groups, the stereotyped perception of internal and external groups, and ethnocentrism. In the field of sports, athletes will anchor sports goals, follow common norms, and seek internal identity according to the overlapping interpersonal boundaries, involved group roles, and similar situational perception characteristics. When the needs of dependent and belonging groups occupy a greater weight in sports decision making, the members’ thinking will tend to the collective nature and characteristics of consistency and similarity, while the actual effect of these results will support individuals to produce values and emotional experiences conducive to the needs of team operation in group activities.

Petrovsky, a former Soviet social psychologist, paid attention to the phenomenon of collectivism in sports. He believed that the sports team is a cooperative group. The relationship between individuals depends on the success or failure of common activities. This group activity itself is the projection of social goals, tasks, and principles [39], reflecting the social consensus formed under the influence of inter group relations. For example, the research results of baseball and football in Japan support the promotion of collectivism culture on competitive sports performance [40]. As an Asian country deeply influenced by Confucian culture, these results in Japan are not surprising. However, PCQ’s research in the field of sports found that American youth athletes also tend to be collectivist in terms of coordination among members [10]. In addition, social identity theory expresses the importance of group influence and process in individual behavior. It believes that individuals define themselves according to their group, while identifying valuable groups can enhance self-esteem and self-concept; at the same time, abiding by group norms is of great significance for individuals to obtain group recognition [41,42]. Brewer (2015) [43] defined it as “the extent to which the in-group has been incorporated into the sense of self, and at the same time, that the self is experienced as an integral part of the in-group”, which is particularly important for a successful sports performance. Recent studies have found that athletes who have a strong perception of the interdependence between results and teammates may also have a stronger sense of social identity for their team [42]. Considering the support for belonging needs, this social identity may further affect the emotion and behavior of team sports members, and lead to a tendency to collectivize. Some studies on personality psychology believe that collective-oriented individuals tend to provide more emotional, information, and evaluation support to others, and to show higher teamwork behavior [20]. These individuals establish their identity on the basis of group members and attribute great value to interdependence, which is common in countries with collectivism as cultural characteristics [23], because collectivism-oriented culture focuses on people’s interdependence, social embeddedness, and obligations and loyalty to internal groups (such as families). Team sports may provide an ideal environment for collective-oriented individuals to meet their desire for collectivity.

Thus, psychological collectivism is the embodiment of high-quality cohesion. The psychological collectivism in team sports is not only affected by the social and cultural background on personality, but also includes the community phenomenon of emotional psychology in small group problems. When the desire to belong to the group occupies a greater weight in sports decision making, psychological collectivism will support individuals to produce emotion and engagement.

Cohesion has a positive effect on athlete engagement through mental toughness. Under the influence of primary groups and someone prestigious, the tolerance of a single person to the current situation can be increased from 21% to 57% [39]. Zhang (2000) believes that athletes in the group environment can be affected by the emotional infection and behavior of other members of the group at any time, and everyone will conform, obey, or depersonalize under the action of special normative factors of the group [5]. Cohesion can not only help the individual members rebound from the pressure to mentality achieve reorganization, but it also can amplify energy and drive the individual members to make continuous efforts toward their peak playing condition. It helps individual members to draw more information, emotion, and instrumental support from the team [44]. These resources help athletes fulfill their sports needs, slow down the setting of self-obstacles, and reduce the potential negative feedback, to activate the athletes’ positive and lasting emotional cognition and obtain athlete engagement.

Cohesion plays a positive role in athlete engagement through psychological collectivism and mental toughness. This study indicates that, in team sports, psychological collectivism is a psychological tendency influenced by the collective environment (cohesion). Under this tendency (psychological collectivism), athletes will improve their mental ability (mental toughness) and then affect their sports performance (athlete engagement). The process can be explained as follows: in a stable sports team structure, the sports team may have the perception of similarity and closeness around group tasks and social interaction. This process is a team goal, personal role, and good interpersonal relationship formed through task and social integration. On this basis, group norms, social identity, and cultural activation may affect the emotion and behavior of sports members who tend to collectivize further. When the desire of belonging to the team occupies a greater weight in sports decision making, individual members will define their preference, reliance, concern, acceptance, and goal priority in competitive sports according to their team, which is to form psychological a collectivism identity for inner groups. This identification makes members have a stronger self-confidence and sense of responsibility in the face of pressure and challenges. At the same time, relying on the social support and response resources established by the group environment, it can also effectively resolve all types of physical and psychological discomfort in the process of sports, realize the balance between the athletes and the environment, and obtain high-quality athlete engagement.

## 6. Conclusions

The four hypotheses proposed in this study were supported. The total score of cohesion and all dimensions can significantly and positively predict athlete engagement, among which ATG-T plays an important role in predicting athlete engagement. Cohesion can affect athlete engagement through a direct and indirect path. The indirect path comprises the intermediary role of psychological collectivism, the intermediary role of mental toughness, and the sequential intermediary role of psychological collectivism and mental toughness. The intermediary effect model constructed in this study has a good fit, which explains 50.5% of the overall variation of athlete engagement, and reveals the mechanism of cohesion on athlete engagement in Chinese team sports to a certain extent.

### 6.1. Implications

First, athlete engagement can be used as an alternative index of sports performance to appropriately reflect the mechanism of cohesion on individual sports performance from a positive psychology perspective. Compared with sensory indicators, athlete engagement can intuitively reflect the positive experience of individual cognition and behavior. Second, this study contributes to the existing literature by examining the relationship between cohesion and psychological collectivism in more depth, in which psychological collectivism is an important feature of a cohesive sports group. Third, the influence of cohesion on athlete engagement needs to be mediated by some type of stress-resistance ability, in which mental toughness, as an inherited or developed advantage, plays an important role.

In Chinese team sports, psychological collectivism is the embodiment of high-quality cohesion. The increase in cohesion and psychological collectivism can improve Chinese athletes’ ability to cope with sports stress situations, which may allow them to achieve a better performance through athlete engagement.

### 6.2. Limitations and Future Study

Although cross-sectional studies can provide valuable information, such studies cannot determine causality. Longitudinal tracking and experimental design may be used to test the findings of this study in the future.

The priming effect of psychological collectivism in team cohesion is not clear. The theories of implicit cultural and social identity are not enough to show that psychological collectivism is a clear turning point in the process of team building. Most studies believe that coaches and athlete leaders are often viewed as responsible for initiating certain strategies targeted toward improving the group environment. Future research can focus on the coach–athlete relationship and the role of leadership in the formation of psychological collectivism in sports groups.

This study only examined the mediating role of psychological collectivism and mental toughness between cohesion and athlete engagement, and failed to involve other influencing factors, such as team size, formal/informal groups, leadership and member roles, team performance, and reference groups. These factors should be further explored in the future.

This study adopted the project packaging strategy, regarded the athlete engagement as an integral variable, and failed to investigate whether the intermediary model has the same effect on all of the dimensions of athlete engagement. This can be further refined in the future, and the project background cam be taken into account to provide targeted help for sports practice.

The findings were generated based on a sample of Chinese athletes, wherein caution needs to be taken when generalizing the results to other populations. Future studies can utilize a random sampling strategy and collect data from various culture backgrounds.

## Figures and Tables

**Table 1:** Reliability and validity analysis.

Variables	Variables	Items	Cronbach’s α	CR	AVE	FL
Cohesion	ATG-T	3	0.77	0.79	0.56	0.72–0.80
	ATG-S	4	0.83	0.84	0.56	0.71–0.79
	GI-T	4	0.77	0.71	0.51	0.49–0.81
	GI-S	3	0.71	0.70	0.44	0.40–0.50
Psychological Collectivism	preference	3	0.90	0.78	0.58	0.56–0.74
	reliance	3	0.78	0.88	0.64	0.68–0.90
	concern	3	0.74	0.76	0.59	0.65–0.78
	acceptance	3	0.77	0.86	0.64	0.69–0.86
	goal priority	3	0.90	0.91	0.71	0.75–0.88
Mental Toughness	self-confidence	4	0.73	0.70	0.50	0.43–0.66
	constancy	4	0.75	0.77	0.54	0.60–0.67
	control	4	0.71	0.74	0.51	0.65–0.98
Athlete Engagement	self-confidence	4	0.90	0.90	0.69	0.78–0.86
	dedication	4	0.90	0.90	0.69	0.76–0.88
	vigor	4	0.91	0.91	0.71	0.78–0.89
	enthusiasm	4	0.92	0.92	0.75	0.83–0.91

**Table 2:** Descriptive statistics of model variables and correlations among model variables.

Component	M	SD	1	2	3	4
Cohesion	4.06	0.52	--			
Psychological Collectivism	3.97	0.63	0.64 ***	--		
Mental Toughness	3.36	0.32	0.35 ***	0.49 ***	--	
Athlete Engagement	4.04	0.63	0.62 ***	0.67 ***	0.43 ***	--

Note: *** *p* < 0.001.

**Table 3:** Cohesion constructs predicting relative contribution of athlete engagement.

Multidimensional Dimension of Cohesion	R^2^	Value-Added Contribution			
		X_1_	X_2_	X_3_	X_4_
—	—	0.352	0.374	0.081	0.333
X_1_	0.352	—	0.041	0.010	0.049
X_2_	0.374	0.019	—	0.010	0.046
X_3_	0.081	0.281	0.303	—	0.260
X_4_	0.333	0.068	0.087	—	0.008
X_1_X_2_	0.393	—	—	0.007	0.033
X_1_X_3_	0.362	—	0.038	—	0.043
X_1_X_4_	0.401	—	0.015	0.004	—
X_2_X_3_	0.384	0.016	—	—	0.040
X_2_X_4_	0.420	0.006	—	0.004	—
X_3_X_4_	0.341	0.064	0.083	—	—
X_1_X_2_X_3_	0.400	—	—	—	0.029
X_1_X_2_X_4_	0.426	—	—	0.003	—
X_1_X_3_X_4_	0.405	—	0.024	—	—
X_2_X_3_X_4_	0.424	0.005	—	—	—
X_1_X_2_X_3_X_4_	0.429	—	—	—	—
Relative importance analysis	—	0.079	0.173	0.034	0.142
Predicted variance percentage	—	18.41	40.33	7.93	33.10

Note: X1, X2, X3, X4 indicate ATG-T, ATG-S, GI-T, GI-S.

**Table 4:** Effect analysis of latent variables.

Influence Path	Standardized Effect Value	Significance	%
Cohesion→Athlete Engagement	0.10	***	14.5
Cohesion→Psychological Collectivism→Athlete Engagement	0.69 × 0.19 = 0.13	***	18.8
Cohesion→Mental Toughness→Athlete Engagement	0.16 × 0.70 = 0.11	***	15.9
Cohesion→Psychological Collectivism→Mental Toughness→Athlete Engagement	0.69 × 0.73 × 0.70 = 0.35	***	50.7
The total indirect effect	0.13 + 0.11 + 0.35 = 0.59	***	85.5
The total effect	0.59 + 0.10 = 0.69	***	— —

Note: *** *p* < 0.001.

## Data Availability

The data in the study are not publicly available in order to protect privacy of the participants.
